# Mangiferin Ameliorates HFD-Induced NAFLD through Regulation of the AMPK and NLRP3 Inflammasome Signal Pathways

**DOI:** 10.1155/2021/4084566

**Published:** 2021-10-25

**Authors:** Zhang Yong, Wang Ruiqi, Yao Hongji, Ma Ning, Jiang Chenzuo, Zhou Yu, Xia Zhixuan, Liu Qiang, Liu Qibing, Lu Weiying, Zhang Xiaopo

**Affiliations:** ^1^Department of Pharmacology, Hainan Medical University, Haikou, Hainan 571199, China; ^2^Key Laboratory of Tropical Translational Medicine of the Ministry of Education, Hainan Key Laboratory for Research and Development of Tropical Herbs, School of Pharmacy, Hainan Medical University, Haikou 571199, China; ^3^Reproductive Medical Center, Hainan Women and Children's Medical Center, Haikou 570206, China

## Abstract

Nonalcoholic fatty liver disease (NAFLD) is closely related to glycolipid metabolism and liver inflammation. And there is no effective drug approved for its clinical therapy. In this study, we focused on mangiferin (Man) and explored its effects and mechanisms on NAFLD treatment based on the regulation of glycolipid metabolism and anti-inflammatory *in vivo* and *in vitro*. The results exhibited that Man can significantly attenuate liver injury, insulin resistance, and glucose tolerance in high-fat diet- (HFD-) induced NAFLD mice and significantly reduce fat accumulation and inflammation in hepatic tissue of NAFLD mice. The transcriptome level RNA-seq analysis showed that the significantly different expression genes between the Man treatment group and the HFD-induced NAFLD model group were mainly related to regulation of energy, metabolism, and inflammation in liver tissue. Furthermore, western blots, real-time PCR, and immunohistochemistry experiments confirmed that Man significantly activated the AMPK signal pathway and inhibited NLRP3 inflammasome activation and pyroptosis in NAFLD mice. In *in vitro* cell experiments, we further confirmed that Man can promote glucose consumption and reduce intracellular triglyceride (TG) accumulation induced by free fatty acids in HepG2 cells and further that it can be blocked by AMPK-specific inhibitors. Western blot results showed that Man upregulated p-AMPK*α* levels and exhibited a significant AMPK activation effect, which was blocked by compound C. At the same time, Man downregulated the expression of NLRP3 inflammasome-related proteins and inhibited the activation of NLRP3 inflammasome, alleviating cell pyroptosis and inflammation effects. These results indicate that Man anti-NAFLD activity is mediated through its regulation of glucolipid metabolism by AMPK activation and its anti-inflammatory effects by NLRP3 inflammasome inhibition. Our study indicates that Man is a promising prodrug for the therapy of NAFLD patients.

## 1. Introduction

As we know, excessive lipid accumulation and steatosis in hepatocytes are the two main characters for nonalcoholic fatty liver disease (NAFLD) patient [1]. NAFLD, as the most commonly liver-related disease, includes simple fatty liver, steatohepatitis, fibrosis, cirrhosis, and even hepatocellular carcinoma [2], which also causes a huge human health challenge and global medical burden [3]. In recent years, with continuous exploration and scientific improvement, the proposed pathogenesis of NAFLD has developed from the “two hit” theory to the “multiple parallel hits” hypothesis, which includes factors such as fat accumulation, lipotoxicity, oxidative stress, inflammation, insulin resistance, endoplasmic reticulum stress, and mitochondrial dysfunction [4–6]. Liver glucose and lipid metabolic disorder and inflammation are the most common initial predisposing factors for NAFLD development [7]. However, the exact pathogenesis is still not fully elucidated, and no effective drug has been given a licence by the FDA to NAFLD treatment in clinical settings. Discovering and investigating effective therapeutic drugs for NAFLD is an urgent problem worldwide.

Glucose and lipid metabolic disorder in hepatocytes is considered to be one of the major NAFLD development risk factors and continually influences the progression of NAFLD. Regulating hepatocyte glucose and lipid metabolism can promote the prevention and treatment of NAFLD [8]. Many enzymes involved in glycolipid metabolism regulation [9]. AMP-activated protein kinase (AMPK), as a cell energy sensor, is one of the key regulatory enzymes for cellular glycolipid metabolism [10]. The metabolic effects of AMPK activation, such as the promotion of glucose consumption, glucose uptake and fatty acid oxidation, inhibition of lipid synthesis and glucose output, and especially its aptitude to arouse a change from fat synthesis to fat oxidation, would be expected to benefit patient with NAFLD, obesity, and type 2 diabetes [10–13]. Targeting AMPK activation is an effective strategy in the exploration of NAFLD treatment based on its regulatory effects on glucose and lipid metabolism.

Inflammation is another key risk factor for NAFLD, especially inflammation caused by lipotoxicity [14, 15]. NLRP3 is the most extensively studied Nod-like receptor associated with inflammation, which forms an inflammasome with its adaptor proteins, and can be activated by many danger factors from surroundings in hepatocyte, including lipotoxicity [14], mitochondrial reactive oxygen species (ROS) [16], and potassium efflux [17]. With activation of NLRP3 inflammasome by danger signals, the caspase-1 is activated and further processes the proinflammatory cytokines interleukin-1*β* (pro-IL-1*β*) and pro-IL-18 and causes the release of mature inflammatory cytokines [16]. Activated caspase-1 also cleaves gasdermin D (GSDMD) to remove autoinhibition of its N-terminal domain (GSDMD-N) [18]. The GSDMD-N further triggers pyroptosis and promotes secretion of IL-1*β* and IL-18 and then causes an inflammatory cascade [18]. The NLRP3 inflammasome and its associated pyroptosis have been proved to play a vital role in NAFLD development in animal models, especially steatohepatitis [19–21]. Suppression of inflammation by inhibiting the NLRP3 inflammasome and pyroptosis is one potential effective therapeutic strategy for NAFLD.

Mangiferin (C2-*β*-D-glycopyranosyl-1,3,6,7-tetrahydroxyxanthone; Man) is a naturally occurring carbon glycoside molecule and is particularly abundant in the leaves of mango, a tropical medicinal plant. Man has a rich biological activity in animal models, such as antioxidative [22], anti-inflammatory [23], anticancer [24], and antidiabetic [25] activity. In addition, a double-blind randomized controlled clinical trial showed that Man significantly reduced triglyceride (TG) and free fatty acid (FFA) levels in serum and improved lipid levels in overweight hyperlipidemia patients [26]. These results suggest that Man is a potential treatment for NAFLD. One recent research has suggested that GSDMD can promote inflammation and pyroptosis in hepatocytes and accelerate the development of NAFLD [27]. Thus, studies on Man could provide new insights for the prevention and treatment of NAFLD. In this study, we study on the effects of Man in the prevention and treatment of NAFLD and explore its mechanisms of glycolipid metabolism regulation and its anti-inflammatory activity based on the AMPK and NLRP3 inflammasome signal pathways.

## 2. Materials and Methods

### 2.1. Animal Experiments

The male C57BL/6J mice, 5 weeks old, were purchased from Vital River Laboratory Animal Technology Co., Ltd. (Beijing, China). Animal experiments were performed at the Animal Research Center of Hainan Medical University under the National Institutes of Health regulations. Our protocols were ratified by the medical ethics committee of Hainan Medical University. The mice were housed at the Animal Research Center of Hainan Medical College under typical environmental surroundings at 25 ± 0.5°C and 12 h light-dark cycle with ad libitum access to standard laboratory water and food.

All mice were randomly divided into 5 groups (*n* = 8 per group): in the normal group, mice were kept with basal diet; in the NAFLD model group, mice were kept with high-fat diet (HFD); in the Man-L group, mice were kept with HFD and administered 25 mg/kg/d Man, i.g.; in the Man-M group, mice were kept with HFD and administered 50 mg/kg/d Man, i.g.; and in the Man-H group, mice were kept with HFD and administered 100 mg/kg/d Man, i.g. The normal group and NAFLD model group mice were administered equal volumes of saline solution. The body weight and blood-glucose concentration after overnight fasting were tested once a week in all animals. After a 12-week treatment, all mice had an absolute diet for 8 h and had taken the whole blood samples from the orbits. Then, the serum samples were got from whole blood for further assay. After blood samples were taken, animals were euthanized with CO_2_ and tissue samples were taken for further analysis.

### 2.2. Oral Glucose Tolerance Test (OGTT) and Insulin Tolerance Test (ITT)

At the last treatment week, the OGTT and ITT were performed, respectively, as follows: (1) OGTT: after fasting overnight, the blood glucose levels were detected and defined as the 0 min time point blood glucose. 2 g/kg of glucose was given orally to all mice, and the concentration of blood glucose was detected at 30, 60, 120, and 180 min time points. (2) ITT: the blood glucose detected after 6 h of fasting, which was defined as the 0 min time point blood glucose. 0.5 U/kg of insulin was then administered by intraperitoneal injection to all mice, and the concentration of blood glucose was detected at 15, 30, 60, and 120 min time points after administration. The blood glucose levels were detected by glucose meter (Roche, ACCU-CHEK) for all glucose tests in this study.

### 2.3. Biochemical Analysis

The levels of serum TG, total cholesterol (TC), low-density lipoprotein cholesterol (LDL-c), high-density lipoprotein cholesterol (HDL-c), alanine aminotransferase (ALT), and aspartate aminotransferase (AST) were tested by appropriate kits from Beijing Strong Biotechnologies, Inc. The serum IL-1*β* level was assayed by IL-1*β* ELISA kit (Beyotime Biotechnology, Nanjing, China).

### 2.4. Liver Histopathological Examination

A section of liver tissue from each mouse was excised from the same part of liver and fixed in 10% formalin for 48 h. The samples were then gone through paraffin embedding, histological section (4 *μ*m), and hematoxylin and eosin (H&E) staining, taken pictures under a light microscope. Pathological liver changes were evaluated and scored by steatosis with 5 grades as follows: zero score is less than 5% of parenchyma involved, one score is between 5% and 25%, two score is between 25% and 50%, three score is between 50% and 75%, and 4 score is over 75%.

### 2.5. RNA-Seq Assay

Four mouse livers were isolated from the model group and the Man-H treatment group, respectively, after the experiment has finished. Total RNA from each mouse liver was extracted and its integrity was assessed. The RNA was then subjected to cDNA library construction, further evaluated the quality of cDNA library by the Agilent Bioanalyzer 2100 system. The clustering of the index-coded samples was performed on a cBot Cluster Generation System using TruSeq PE Cluster Kit v3-cBot-HS (Illumina) according to the manufacturer's instructions. After cluster generation, the library preparations were sequenced on an Illumina Novaseq platform and 150 bp paired-end reads were generated. Pearson correlations were performed between samples. The difference gene, Gene Ontology (GO), and Kyoto Encyclopedia of Genes and Genomes (KEGG) assays were performed for the RNA-seq results.

### 2.6. Immunohistochemical Assay

In brief, immunohistochemical assays were conducted on 4 *μ*m liver sections after rehydration and antigen retrieval. The sections were first incubated with blocking buffer, then exposed to the primary antibody at 4°C for 12 h, followed by TBS washing 3 times and incubation with a properly diluted secondary antibody for 1–2 h at 25°C. Sections were washed with TBS 3 times and stained with diaminobenzidine substrate solution peroxidase substrate kit (Beyotime Biotechnology, Nanjing, China). All immunohistochemical analyses were repeated at least 3 times, and representative images were captured and presented.

### 2.7. Cell Culture and Treatment

HepG2 cells were cultured with Dulbecco's modified Eagle medium containing 10% fetal bovine serum (FBS) and appropriate antibiotics at 37°C in a cellular incubator with 5% CO_2_. Man (Biopurify Phytochemicals Ltd., lot number B20837, purity ≥ 99%) and metformin (Met) (Shanghai Yuanye Bio-Technology Co., Ltd., lot number 20191008, purity ≥ 98%) were all dissolved in dimethylsulfoxide (DMSO). Met was used as the positive control for cellular experiments. The cells were seeded into cell culture plates (96/24/6-well) and cultured for approximately 24 h. Study drugs were added after overnight starvation in 0.5% FBS-containing medium.

### 2.8. Cytotoxicity Assay

The cell viability was evaluated by the MTT method. First, the HepG2 cells were seeded into 96-well plates at 2.0 × 10^4^ cells per well. After 24 h of incubation and 8 h of serum starvation, Man was added at the specified concentration treatment for 24 h and each treatment with 5 replicates. Control treatment wells were added DMSO as equal concentration of 400 *μ*M of Man treatment wells. Then, the media supernatants were discarded and cells were added with 50 *μ*l MTT solution, incubated it at 37°C for 4 h. The OD values at 490 nm were read at a microplate reader for formazan level detection. Results were exhibited as percentages of controls wells, normalized to 100.

### 2.9. Glucose Consumption Assay

The HepG2 Cells cultured and seeded as above description. The cells were treated with DMSO, Man, or Met at the indicated concentrations for 24 h with 5 replicates. The glucose level of cellular culture supernatant was tested by the Randox glucose assay kit (Beijing Strong Biotechnologies, Inc.). The base glucose consumption of each compound treatment was calculated as glucose level of the fresh medium minus the cultured medium glucose level.

### 2.10. Intracellular Triglyceride Assay

The palmitic acid (PA) coupled with bovine serum albumin (BSA) method was described previously [28]. First, the fatty acid-free BSA was added to DMEM medium to achieve a 10% BSA solution. The PA powder then was added to 10% BSA solution to get a 7.5 mM solution of PA coupled with BSA (PA-BSA). The HepG2 cells were cultured and seeded as above description. After starving overnight, PA-BSA was added to wells at 300 *μ*M concentration. The 10% BSA solution treatment was used as a control and performed at the same time. After 24 h incubation, PA-BSA, DMSO, Man, and Met at the indicated concentration were then added to wells for another 24 h incubation. The levels of intracellular TG were detected by tissue TG assay kit (Applygen Technologies Inc.) and normalized to sample protein concentrations.

### 2.11. Oil Red O (ORO) Staining

We further evaluate the intracellular lipid accumulation induced by PA-BSA in hepatocyte by ORO staining. Briefly, HepG2 cells were fixed with 10% formalin for 1 hour and stained then with ORO solution for 45 min. The cells were washed with deionized water and observed under a light microscope.

### 2.12. Real-Time PCR Assay

Total RNA was extracted from HepG2 cells and mouse liver tissues after treatment. The cDNA were made by the commercial kit per its protocols, which were used as the templates of real-time PCR. Real-time PCR was performed in an ABI PRISM 7900 High-Throughput Real-Time RCR System. The forward (F) and reverse (R) primer sequences were as follows: NLRP3 (F, 5′-tcacaactcgcccaaggaggaa-3′; R, 5′-aagagaccacggcagaagctag-3′); caspase-1 (F, 5′-acaaggcacgggacctatg-3′; R, 5′-tcccagtcagtcctggaaatg-3′); IL-1*β* (F, 5′-cgacaaaatacctgtggcct-3′; R, 5′-ttctttgggtattgcttggg-3′); and *β*-actin (F, 5′-ggatgcagaaggagattactgc-3′; R, 5′-ccaccgatccacacagagta-3′).

### 2.13. Western Blot

Total proteins were extracted from cells or liver tissues. Western blots for each sample were produced as follows: samples with 50 *μ*g total protein were added to 10% or 12% sodium dodecyl sulfate-polyacrylamide gel electrophoresis (SDS-PAGE) and protein bands were electrically transferred to a PVDF membrane (Merck, Germany). After 5% skimmed milk blocking overnight at 4°C, detections of phospho-AMPK*α* (Thr172) (p-AMPK*α*), AMPK*α*, phospho-ACC (Ser79) (p-ACC), ACC, NLRP3, caspase-1, caspase-1 (p10), IL-1*β*, GSDMD-N, and *β*-actin proteins were achieved with specific monoclonal primary and secondary antibodies. The protein bands were developed by ECL kit (Beyotime Biotechnology, Nanjing, China). The blots were scanned and quantified, levels of p-AMPK*α* and p-ACC were normalized to that of AMPK*α* and ACC, respectively, and NLRP3, caspase-1, caspase-1 (p10), IL-1*β*, and GSDMD-N were normalized to that of *β*-catenin. The level of target protein was presented as fold of control treatment.

### 2.14. Blocking Experiments

HepG2 cells were cultured and seeded as above. Before drug treatment, the cells were pretreated with compound C (dissolved in DMSO) at 10 *μ*M for 1 h, and DMSO was added to the untreated wells. Following pretreatment, the drugs were added to corresponding wells and cocultured with the cells for 24 h. Total cellular protein was extracted after treatment and detected by western blot. In parallel experiments, cell culture supernatants were centrifuged at 500 *g* for 10 min, and glucose consumption of every treatment was tested by glucose assay kits as described above. In other parallel experiments, intracellular TG was detected by TG assay kit as described above and normalized to protein concentration from each treatment.

### 2.15. Statistical Analysis

All values from *in vitro* experiments are presented as the mean ± SD, and each experiment was replicated at least 3 times; all values from *in vivo* experimental results are shown as the mean ± SD; 8 mice were included in each group. Data processing and statistical analysis were used by GraphPad Prism 5.0 software. One-way ANOVA was used to detect the differences among studied groups. *p* < 0.05 is considered to be statistically significant.

## 3. Results

### 3.1. Man Reduces Weight and Lowers Blood Glucose and Blood Lipids in HFD-Induced NAFLD Mice

**Figure 1:**
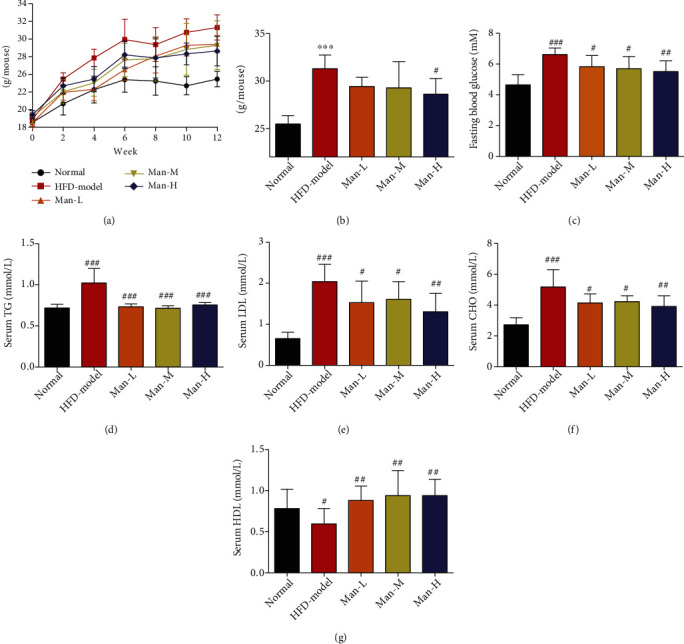
Man reduces weight and lowers blood glucose and blood lipids in HFD-induced NAFLD mice. (a) The body weight change of mice during experimental period. (b) The body weight of mice after a 12-week treatment. (c) The last FBG before the finished experiment. (d–g) Serum levels of TG (d), LDL-c (e), CHO (f), and HDL-c (g). The results represent as the mean ± SD, *n* = 8. ^∗^*p* < 0.05, ^∗∗^*p* < 0.01, and ^∗∗∗^*p* < 0.001 vs. the normal group. ^#^*p* < 0.05, ^##^*p* < 0.01, and ^###^*p* < 0.001 vs. the model group.

After 12 weeks of HFD, the weight of the model group NAFLD mice (31.32 ± 1.43 g) was obviously higher than that of the normal group (25.47 ± 0.87 g) (Figures 1(a) and 1(b), *p* < 0.001), and the levels of fasting blood glucose (FBG) (6.61 ± 0.42 mM), TG (1.02 ± 0.17 mM), LDL-c (2.04 ± 0.42 mM), and CHO (5.18 ± 1.12 mM) were more higher than those of the normal control group (FBG: 4.65 ± 0.66 mM; TG: 0.71 ± 0.04 mM; LDL-c: 0.65 ± 0.15 mM; CHO: 2.72 ± 0.45 mM) significantly, while the level of HLD-c (0.59 ± 0.18 mM) was lower than that of the normal group (0.78 ± 0.23 mM) significantly (Figures 1(d)–1(g), *p* < 0.001). After the 12-week experimental period, mice from the Man intervention treatment groups weighed significantly less than the NAFLD model group (31.32 ± 1.43 g), especially in the high-dose (Man-H) group (28.63 ± 1.64 g) (Figure 1(b), *p* < 0.05). Man treatment also significantly reduced FBG, improved serum lipid TG, LDL-c, and CHO-c levels (Figures 1(d)–1(g), *p* < 0.05), and exhibited dose dependency. At the same time, Man also increased HLD-c levels, which is conducive to regulating fat metabolism (Figure 1(g), *p* < 0.01).

### 3.2. Man Improves Hepatic Fatty Deposition and Reduces Liver Damage

**Figure 2:**
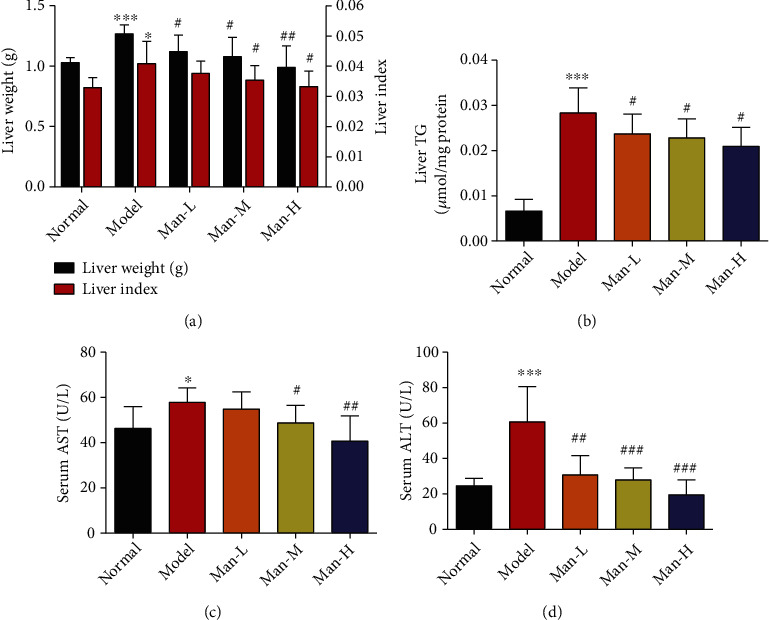
Man improves hepatic fatty deposition and reduces liver damage. (a) Liver weight and liver index of animals. (b) TG levels of liver. (c, d) The levels of AST and ALT in serums. All results represent as the mean ± SD, *n* = 8. ^∗^*p* < 0.05, ^∗∗^*p* < 0.01, and ^∗∗∗^*p* < 0.001 vs. the normal group. ^#^*p* < 0.05, ^##^*p* < 0.01, and ^###^*p* < 0.001 vs. the model group.

In the HFD-induced NAFLD model mice, liver weight and liver index were obviously increased compared to that in the normal group mice (Figure 2(a), *p* < 0.001 or *p* < 0.05). At the same time, the liver TG was also significantly increased in NAFLD mice compared to the normal control group (Figure 2(b), *p* < 0.001), suggesting that hepatomegaly may be caused by liver TG accumulation in NAFLD model. However, for mice in the Man-M and Man-H groups, liver weight and liver index were significantly decreased compared to the model group (Figure 2(a), *p* < 0.05), and all doses of Man treatment inhibited liver TG increases (Figure 2(b), *p* < 0.05). Moreover, the levels of liver functions index of ALT and AST in the NAFLD model group mouse serum markedly increased compared to those in the normal group mice (Figures 2(c) and 2(d), *p* < 0.05), suggesting that excessive TG accumulation may cause liver injury. However, the levels of AST and ALT in the Man treatment group mouse serum were significantly decreased compared to those in the no-treatment model group (Figures 2(c) and 2(d), *p* < 0.05), suggesting that Man could reduce liver damage from adipopexis.

### 3.3. Man Improved Insulin Resistance and Glucose Tolerance in NAFLD Mice

**Figure 3:**
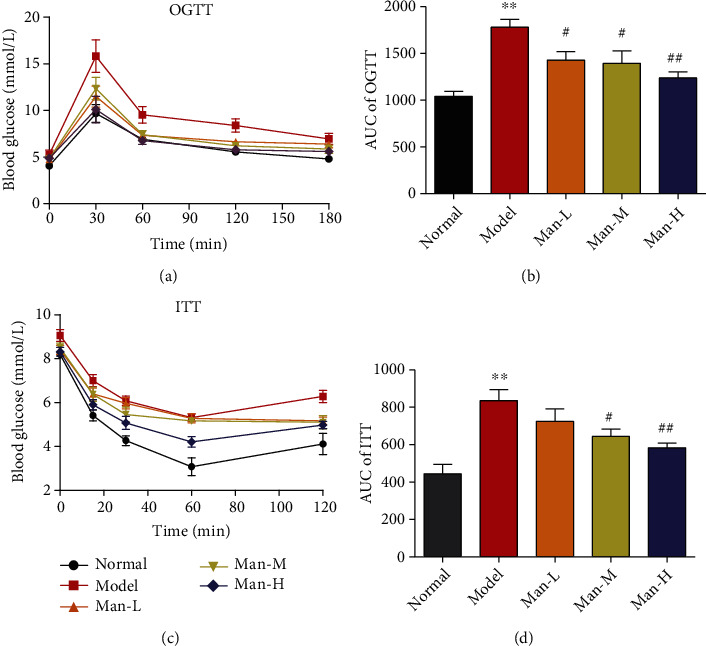
Man ameliorates insulin resistance and glucose tolerance in NAFLD mice. (a) The curve of OGTT. (b) The area under the curve of OGTT. (c) The curve of ITT. (d) The area under the curve of ITT. Data represent as the mean ± SD (*n* = 8). ^∗∗^*p* < 0.01 vs. that of the normal group. ^#^*p* < 0.05 and ^##^*p* < 0.01 vs. that of the NAFLD model group.

The ITT and OGTT *in vivo* were executed to characterize whether Man could improve hepatic insulin resistance. As compared to the normal group, the HFD-induced NAFLD mouse group showed obvious impaired insulin resistance and glucose tolerance as marked by the important increase in the area under curve (AUC) of OGTT and ITT (Figures 3(a)–3(d), *p* < 0.001 vs. the normal group). After 12 weeks of Man treatment, the AUCs of OGTT and ITT were dose-dependently reduced, suggesting that insulin resistance of NAFLD mice was distinctly improved compared to the NAFLD model group mice (Figures 3(a)–3(d), *p* < 0.01 vs. the NAFLD model group).

### 3.4. Man Improves Hepatic Steatosis of HFD-Induced NAFLD Mice

**Figure 4:**
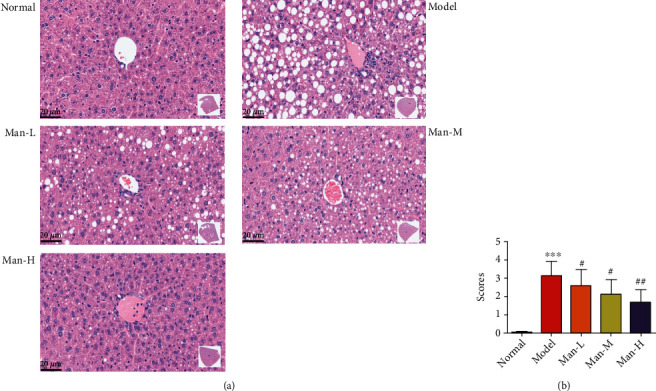
Man improves hepatic steatosis in HFD-induced NAFLD mice. (a) The H&E staining, one representativeness of pathological images of each group. (b) The steatosis scores of liver pathological changes. Data represent as the mean ± SD (*n* = 8). ^∗∗∗^*p* < 0.001 vs. that of the normal group. ^##^*p* < 0.01 vs. that of the NAFLD model group.

In the liver histopathology examination, serious fatty accumulation and degeneration were also detected in NAFLD model mice, and more diffuse hepatocellular bullae steatosis were observed in liver pathology slice (Figure 4(a)), which may also damage the liver function. And the pathological steatosis scores were more higher than the normal group (Figure 4(b), *p* < 0.05), which indicated that more severe steatosis occurs in the liver of model mice and was effectively improved by Man treatment dose-dependently (Figure 4, *p* < 0.05 vs. the NAFLD model group), and the numbers of hepatocyte involved in liver diffuse bullae steatosis were also decreased seriously (Figure 4(a)). Our results suggest that Man can improve liver steatosis and function in histopathology level *in vivo.*

### 3.5. RNA-Seq Assay of Man's Effects on NAFLD Treatment

**Figure 5:**
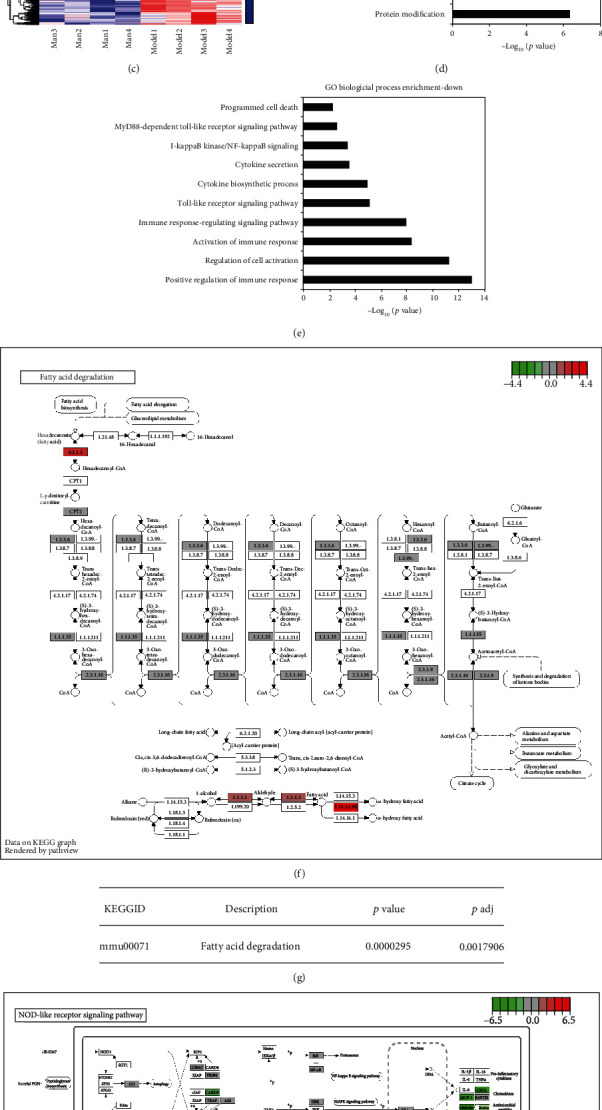
RNA-seq assay of mouse hepatocytes between Man treatment and NAFLD model groups. (a) The Pearson correlation between samples. (b) Differentially expressed gene volcano map (∣log_2_ (fold change)∣>1 and *p*adj ≤ 0.05). (c) Differentially expressed genes clustering heat map of 1601 (∣log_2_ (fold change)∣>1 and *p*adj ≤ 0.05). (d, e) The Gene Ontology (GO) biological process enrichment of upregulation (d) or downregulation (e) of differentially expressed genes, and the threshold value for significant enrichment of GO function was *p*adj ≤ 0.05. (f, g) One of the representations of KEGG pathway (f) about upregulation of differentially expressed genes and its ID, description, *p* value, and *p*adj value (g). (h, i) One of the representations of KEGG pathway (h) about downregulation of differentially expressed genes and its ID, description, *p* value, and *p*adj value (i). (j) The NLRP3/Casp-1/GSDMD-mediated inflammatory signal pathway in (h), which is closely related to the effects of Man treatment.

To explore the potential molecular mechanisms of Man's effects on NAFLD mouse livers, RNA-seq was performed on liver tissue from 4 mice with or without Man (100 mg/kg/d) treatment. The Pearson *R*^2^ values of all Man-treated samples and NAFLD model mouse samples were over 0.92, suggesting that the samples exhibited good repeatability (Figure 5(a)). The 1601 differentially expressed genes (∣log_2_ (fold change)∣>1 and corrected *p* value (*p*adj) ≤ 0.05) were found using the DESeq2 R package (1.20.0) and visualized with volcano plot as shown in Figure 5(b), in which 448 genes were upregulated and 1153 genes were downregulated (Figure 5(b)). The heat map as shown in Figure 5(c) was also used to visualize the differentially expressed genes. The Gene Ontology (GO) enrichment analysis was done by the clusterProfiler R package, and GO terms with corrected *p* value less than 0.05 were considered significantly enriched. The GO biological processes of significantly upregulated genes are shown in Figure 5(d), which were mainly related to energy derivation, cellular respiration, fatty acid metabolic processing, and generation of precursor metabolites and energy, and associated with liver metabolism of energy substances, such as glucose, free fatty acids, and lipids. We used the clusterProfiler R package to test the statistical enrichment of differentially expressed genes in KEGG pathways and found that Man could significantly promote fatty acid degradation signaling pathways by increasing the E6.2.1.3 gene expression (Figure 5(f)), which is one of the key enzymes for fatty acid oxidation (Figure 5(g), *p* value = 0.00003). For the downregulated genes, the GO biological processes were mostly associated with inflammation, such as immune response, the Toll-like receptor signaling pathway, cytokine biosynthetic processing, the NF-KB signaling pathway, and programmed cell death, among others, and the representative 10 biological processes as shown in Figure 5(e). Those results suggested that Man might exhibit anti-inflammatory activity. The KEGG pathway assay of these downregulated genes found that Man could significantly inhibit the nod-like receptor signal pathway (Figures 5(h) and 5(i), *p* value = 0.0019), which mainly downregulated NLRP3 gene expression and regulated the NLRP3/caspase-1/GSDMD signal pathway (Figure 5(j)). All these results showed that Man might possess beneficial effects on energy metabolism and inflammation for treatment of HFD-induced NAFLD mice.

### 3.6. Man Activates AMPK and Inhibits the NLRP3/Caspase-1/GSDMD Signaling Pathway in Hepatocyte *In Vivo*

**Figure 6:**
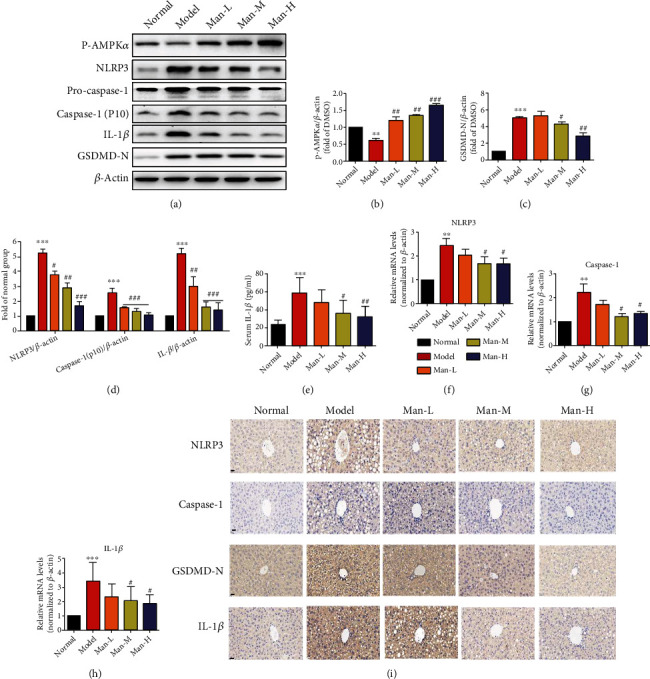
Man activates AMPK and regulates the NLRP3 inflammasome signaling pathway to exert anti-inflammatory effects *in vivo*. The total protein was extracted from animal liver of experiment. The p-AMPK, NLRP3, pro-caspase-1, caspase-1 (p10), IL-1*β*, and GSDMD-N protein levels were detected by Western blots and quantified by using ImageJ software. (a) Western blots. (b) The protein levels of p-AMPK were normalized to those of *β*-actin, respectively, and plotted as fold of the normal group. (c) The protein levels of GSDMD-N were normalized to those of *β*-actin, respectively, and plotted as fold of the normal group. (d) The protein levels of NLRP3, caspase-1 (p10), and IL-1*β* were normalized to those of *β*-actin, respectively, and plotted as fold of the normal group. (f–h) The NLRP3 and ASC caspase-1 mRNA levels were detected by quantitative real-time PCR in animals' liver. The data are expressed as the mean ± SD (*n* = 8). ^∗^*p* < 0.05, ^∗∗^*p* < 0.01, and ^∗∗∗^*p* < 0.001 vs. the normal group. ^#^*p* < 0.05 and ^##^*p* < 0.01 vs. the NAFLD model group.

AMPK is a key regulatory enzyme of energy balance in liver tissue cells, which is associated with hepatocyte glucolipid metabolism and is a potential target for NAFLD therapy. As our RNA-seq assay showed that genes significantly upregulated by Man treatment were mostly associated with energy metabolism (Figure 5(d)), the AMPK activation in mouse hepatocytes was detected by western blot. The results showed that AMPK activity in the NAFLD mouse liver tissues was significantly inhibited, while the level of p-AMPK*α* was potently upregulated in Man treatment mice dose-dependently (Figures 6(a) and 6(b)). Meanwhile, we further investigated the effects of Man on NLRP3-mediated hepatocyte inflammation and pyroptosis. Our results exhibited that liver NLRP3, caspase-1, caspase-1 (p10), and IL-1*β* were significantly upregulated in the NAFLD model group mice (Figures 6(a), 6(c), and 6(d)) and which were significantly decreased in Man-treated mice (Figures 6(a), 6(c), and 6(d)). These suggested that Man can inhibit the activation of NLRP3 inflammasome. We further examined the GSDMD splicing and pyroptosis induced by NLRP3 inflammasome activation and found that Man could inhibit GSDMD splicing and pyroptosis mediated by NLRP3 inflammasome activation (Figures 6(a) and 6(c)), thus playing an anti-inflammatory effect. Through the quantification of serum IL-1*β*, we also found that serum IL-1*β* levels significantly decreased with Man treatment (Figure 6(e), *p* < 0.05). The RT-PCR results demonstrated that Man significantly downregulated the transcription levels of NLRP3, caspase-1, and IL-1*β* genes (Figures 6(f)–6(h), *p* < 0.05). Immunofluorescence assays showed that the positive staining degrees of NLRP3, caspase-1, GSDMD-N, and IL-1*β* in NAFLD mouse liver tissues were visibly high compared to those of the normal control group, indicating upregulation of NLRP3, caspase-1, and GSDMD expression (Figure 6(i)), and Man could reduce the positive staining levels (Figure 6(i)), indicating downregulation of those proteins, which was consistent with the western blot and RT-PCR results.

### 3.7. Man Cytotoxicity in HepG2 Cells

**Figure 7:**
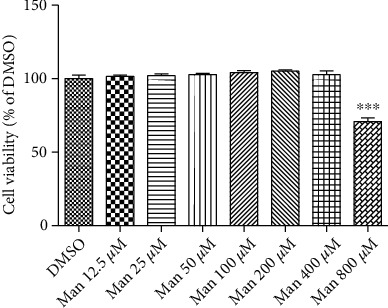
Man effects on cell viability. HepG2 cells were incubated with Man at indicated concentrations for 24 h; cell viabilities were tested by MTT assay. All result values are represented as the mean ± SD (*n* = 5). ^∗∗∗^*p* < 0.001 vs. DMSO.

As shown in Figure 7, 400 *μ*M Man displayed no cytotoxicity with 24 h treatment in HepG2 cells. Importantly, it reduced the viability of HepG2 cells at higher concentrations (800 *μ*M; Figure 7, *p* < 0.05 vs. DMSO).

### 3.8. Man Activates AMPK to Regulate Hepatocyte Glucose and Lipid Metabolism *In Vitro*

**Figure 8:**
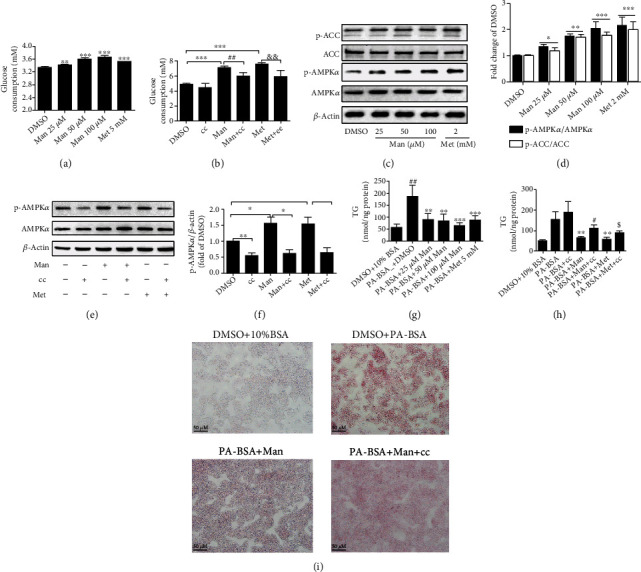
Man modulates cell glycolipid metabolism by activating the AMPK signal pathway. (a) Glucose consumptions. All result values are represented as the mean ± SD (*n* = 5). ^∗∗^*p* < 0.01 or ^∗∗∗^*p* < 0.001 vs. DMSO. (b) Glucose consumptions with compound C pretreatment. Values are represented as the mean ± SD (*n* = 5). ^∗∗∗^*p* < 0.001 vs. DMSO, ^##^*p* < 0.01 vs. Man, and ^&&^*p* < 0.01 vs. Met. (c) After Man treatment, the total cell proteins of every well were extracted; p-AMPK*α* (Thr172) (p-AMPK*α*), AMPK*α*, p-ACC (Ser79) (p-ACC), ACC, and *β*-actin were determined by western blot. Representative blot pictures as presented. (d) Normalizing the level of p-AMPK*α* and p-ACC to those of AMPK*α* and ACC, respectively, and plotted as fold of DMSO-treated cells. The values are represented as the mean ± SD (*n* = 3). ^∗^*p* < 0.05, ^∗∗^*p* < 0.01, and ^∗∗∗^*p* < 0.001 vs. DMSO. (e) HepG2 cells were pretreated with cc for 60 min; then, Man was added and incubated for 24 h; total cell proteins were extracted; p-AMPK*α* (Thr172) (p-AMPK*α*) and AMPK*α* were detected by western blot. Representative blot pictures as presented. (f) The protein level of p-AMPK*α* was normalized to AMPK*α*, respectively, and plotted as fold of DMSO-treated cells. Values are represented as the mean ± SD (*n* = 3). ^∗^*p* < 0.05, ^∗∗^*p* < 0.01, and ^∗∗∗^*p* < 0.001 vs. DMSO. (g) The intracellular TG levels of HepG2 cells were detected after PA-BSA or PA-BSA plus Man or Met treatment for 24 h and normalized to protein content of the sample. All result values are represented as the mean ± SD (*n* = 4). ^∗∗^*p* < 0.01 vs. DMSO+10% BSA and ^##^*p* < 0.01 or ^###^*p* < 0.001 vs. PA-BSA+DMSO. (h) The intracellular TG levels of HepG2 cells were detected and normalized to protein content of the sample, each treatment with 4 replications. Values are represented as the mean ± SD (*n* = 4). ^∗∗∗^*p* < 0.001 vs. 10% BSA+DMSO, ^##^*p* < 0.01 vs. PA-BSA, ^&^*p* < 0.05 vs. PA-BSA+Man, and ^$^*p* < 0.05 vs. PA-BSA+Met. (i) ORO staining.

The effect of Man on glucose metabolism was investigated via glucose consumption assay. As shown in Figure 8(a), Man increases the basal glucose consumption level of HepG2 cells in a dose-dependent manner (Figure 8(a), *p* < 0.01 or *p* < 0.001 vs. DMSO). 25 *μ*M Man significantly increased glucose consumption (Figure 8(a), *p* < 0.01 vs. DMSO), and 100 *μ*M Man was comparable to that of 2 mM metformin (Figure 8(a)). Moreover, its activity was abolished by pretreatment with compound C (cc), an AMPK inhibitor. These results suggest that the effects of Man may be dependent on AMPK activation (Figure 8(b), *p* < 0.01 vs. Man). In fact, our results showed that Man could activate AMPK and dose-dependently increased the level of p-AMPK*α* (Thr172) and p-ACC (Ser79) in HepG2 cells after 24 h of administration (Figures 8(c) and 8(d), *p* < 0.05 or *p* < 0.01 or *p* < 0.001 vs. DMSO). The observed AMPK-stimulating activation of Man was completely blocked by cc (Figures 8(e) and 8(f), *p* < 0.05). To investigate Man effects on lipid metabolism, the hepatocyte of HepG2 cells was induced by PA-BSA to accumulate TG, and Man was administered to prevent PA-BSA-induced intracellular TG accumulation. As shown in Figure 8(g), 0.3 mM of PA-BSA treatment for 24 h increased intracellular TG levels dramatically (Figure 8(g), *p* < 0.01 vs. DMSO+10% BSA), which were dose-dependently reduced by Man (Figure 8(g), *p* < 0.01 or *p* < 0.001 vs. PA-BSA+DMSO). Furthermore, this effect of Man on reducing intracellular TG accumulation was blocked by cc pretreatment for 60 min in HepG2 cells (Figure 8(h), ^&^*p* < 0.05 vs. PA-BSA+Man). ORO staining was performed to further determine Man effects on lipid metabolism. As shown in Figure 8(i), cells treated with PA-BSA and DMSO exhibited more red positive staining, which was significantly reduced by 100 *μ*M Man treatment (Figure 8(i)), but not after cc pretreatment for 60 min before Man treatment compared to Man treatment alone (Figure 8(i)).

### 3.9. Man Inhibits the NLRP3/Caspase-1/GSDMD Signal Pathway in Hepatocytes

**Figure 9:**
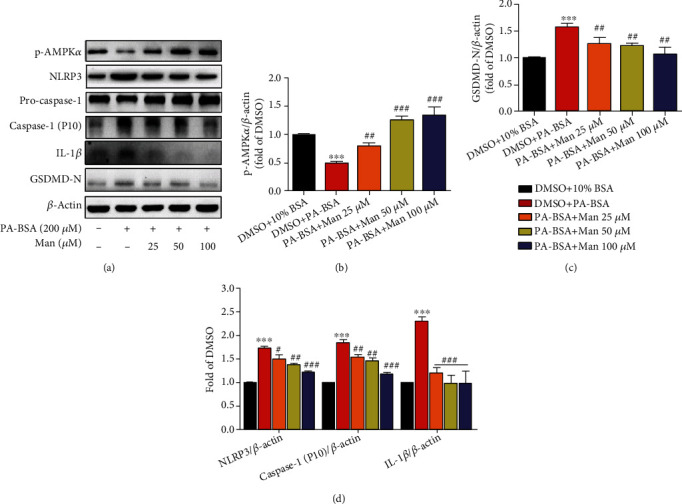
The effects of Man on NLRP3 inflammasome signal axis and its inhibition of NLRP3-mediated pyroptosis *in vitro*. (a) After serum starvation treatment, cells were untreated or treated with PA-BSA or PA-BSA+Man or PA-BSA+Met as indicated for 24 h. Then, total cell proteins were made; p-AMPK*α* (Thr172) (p-AMPK*α*), NLRP3, caspase-1, caspase-1 (P10), GSDMD-N, IL-1*β*, and *β*-actin were detected by western blot. Representative blot pictures as shown. The protein levels of p-AMPK*α* (b), GSDMD-N (c), and NLRP3/caspase-1 (p10)/IL-*β* (d) were normalized to *β*-actin, respectively, and plotted as fold of the DMSO+10% BSA-treated group. Values are represented as the mean ± SD (*n* = 3). ^∗∗∗^*p* < 0.001 vs. DMSO+10% BSA and ^##^*p* < 0.01 and ^###^*p* < 0.001 vs. DMSO+PA-BSA.

The inflammation from NLRP3 inflammasome activation plays a crucial role in many metabolic diseases including NAFLD, and our animal experiments showed that Man inhibits NLRP3-mediated inflammation and pyroptosis in hepatocytes by western blot and RNA-seq analysis. Similar results were obtained in HepG2 cells with free fatty acid (0.3 mM PA-BSA) induction of NLRP3 inflammasome activation, as shown in Figure 9. Compared to the DMSO+10% BSA treatment group, p-AMPK*α* levels were significantly decreased in PA-BSA-treated HepG2 cells (Figures 9(a) and 9(b), *p* < 0.001 vs. DMSO+10% BSA), which were significantly increased by Man (Figures 9(a) and 9(b), *p* < 0.01 or *p* < 0.001 vs. DMSO+PA-BSA). At the same time, the protein levels of NLRP3, caspase-1 (p10), IL-1*β*, and GSDMD-N were obviously increased in PA-BSA-induced HepG2 cells (Figure 9(a)), indicating that FFA (0.3 mM PA-BSA) can activate the NLRP3 inflammasome and cell pyroptosis to induce inflammation. However, mangiferin significantly reversed this phenomenon, reduced the expression of these proteins, inhibited the activation of NLRP3 inflammasomes and pyroptosis, and played an anti-inflammatory effect *in vitro* (Figures 9(c) and 9(d)).

## 4. Discussion

NAFLD is considered to be ultralimit lipid accumulation and steatosis in hepatocytes without ethanol consumption and encompasses a broad disease spectrum including simple fatty liver, steatohepatitis, fibrosis, cirrhosis, and liver cancer [1]. The “two-hit” and “multiple parallel hits” hypotheses had been used to explain NAFLD development, and the latter hypothesis is more widely favored. Even so, the pathogenesis of NAFLD remains unclear and very complex. Risk factors leading to NAFLD development include lipotoxicity, oxidative stress, inflammation, endoplasmic reticulum stress, insulin resistance, and mitochondrial dysfunction, which are also potential therapeutic targets for the development of NAFLD treatments [29]. At the present time, there is no long-term efficacious and approved standard therapy for NAFLD treatment. Clinically, the drugs such as hypoglycemic, lipid-lowering, and insulin sensitizers are usually chosen during the NAFLD treatment; at the same time, diet control and exercise are necessary [10, 29]. In this work, we are interested in the potential anti-NAFLD activity and mechanisms of Man and focusing on its regulation of glycolipid metabolism and anti-inflammatory *in vitro* and *in vivo*. In our experiments, HFD-fed-induced NAFLD mice indicated that Man treatment significantly reversed body and liver weight increases and improved liver index, dyslipidemia, liver damage, insulin resistance, and inflammation. In *in vitro* HepG2 cell experiments, Man significantly increased glucose consumption and decreased intracellular TG levels, lipid accumulation, and inflammation induced by PA-BSA. We further demonstrated that this protective effect of Man might contribute to its regulation effects on the AMPK and NLRP3 inflammasome signaling pathways.

The liver is a vital organ for controlling circulating glucolipid metabolism and plays a key role in the progress of NAFLD, T2D, and atherosclerosis [13]. AMPK is a highly conserved kinase that regulates energy balance, and is thought as an important and interesting target for the treatment of many glucose and lipid metabolic disorder diseases, such as NAFLD, diabetes, dyslipidemia, and obesity [13, 30, 31]. Activating liver AMPK can promote glucose metabolism and lipid oxidative decomposition to provide energy for the body [12]. AMPK agonists, such as metformin, berberine, and gastrodin, were considered potential drugs used for the treatment of a variety of diseases related to glucose and lipid metabolism disorders, including NAFLD, diabetes, and cardiovascular and cerebrovascular diseases [13, 31]. Our results show that Man significantly activates AMPK of hepatocytes with *in vitro* and *in vivo* experiments. Man accelerates HepG2 cell basal glucose consumption and reduces TG accumulation induced by PA-BSA, and its effects are abolished by an AMPK-specific inhibitor, compound C. Our ORO staining test also confirmed the lipid-lowering effect of Man and its dependence on AMPK activation. In the HFD-induced NAFLD animal experiments, we also observed the hypoglycemic and lipid-lowering effects of Man in serum. Liver histopathological and transcriptomic analyses also showed that Man significantly reduced fat storage and degeneration in liver tissue. Previous studies showed that Man reduces weight and lowers glucose and lipid in a diabetic rat model [32], which is consistent with our results. Obesity is also a common cause of NAFLD development, and several studies showed that AMPK activation is a potential therapeutic target for weight loss drugs [33, 34]. Our study showed that Man also has a weight loss effect in HFD-induced NAFLD model mice. Thus, AMPK activation by Man may be one of the key mechanisms of its anti-NAFLD effects. The AMPK signal pathway is mainly activated by two different ways, one is Ca^2+^-dependent pathway regulation by CaMKK*β* and another is AMP-dependent pathway mediated by LKB1, and the exact molecular mechanism of how Man activates AMPK is our focus for future work.

Inflammasomes are multiprotein complexes that mediate the production of inflammatory cytokines and produce potent inflammatory cascades [35]. The NLRP3 inflammasome is the most widely studied and characterized member of this family. As extensively reported, the NLRP3 inflammasome mediates inflammation induced by many noninfectious factors, such as lipid accumulation, oxidative stress from hyperglycemia, and hyperlipidemia [35, 36], and participates in the causing of many diseases, including NAFLD, obesity, diabetes, and cardiovascular diseases [16, 35, 37]. Many inflammasome inhibitors, such as isoliquiritigenin and GS9450, may be potential anti-NAFLD drugs. In our study, we confirmed that Man inhibits NLRP3 inflammasome activity at both the cellular and organism levels through a series of experiments. First, Man significantly downregulated the levels of NLRP3, caspase-1, and IL-1*β* in the liver tissues of HFD-induced NAFLD mice as measured by western blot and RT-PCR. At the cellular level, Man significantly decreased the levels of NLRP3, caspase-1, and IL-1*β* induced by PA-BSA treatment of HepG2 cells. Liver tissue immunohistochemical analysis also exhibited that Man distinctly inhibits NLRP3 inflammasome function, decreased inflammatory factor production and release, and then suppressed the occurrence of inflammation. At the same time, we also analyzed transcriptional sequencing and observed that the Man-treated groups exhibited significantly downregulated transcription of genes related to the biochemical process of inflammation. The KEGG pathway assay also showed that Man had a significant impact on the NLRP3 inflammasome signaling pathway. All these results indicated that Man exerts anti-inflammatory effects by inhibiting the activation of NLRP3 inflammasomes, which may be an important effect on NAFLD treatment.

Our study also found that Man can improve insulin resistance in HFD-induced NAFLD mice. Insulin resistance is also a common risk factor for the occurrence and development of NAFLD, especially in type 2 diabetic patients. It is recognized that improving insulin resistance can significantly improve the progression of NAFLD [38], and it is currently a common method for the clinical treatment of NAFLD. Others studies have reported that Man improves insulin resistance in diabetic mice, and we also observed a significant improvement in insulin resistance in NAFLD models [39]. This may be related to its ability to activate AMPK in hepatocytes because studies have shown that AMPK activation can improve insulin resistance, but the specific mechanism needs to be further studied. In addition, we also observed protective effects of Man on liver function, which may be associated with improved insulin resistance and glucose tolerance, and have a positive effect on the prevention and treatment of NAFLD.

## 5. Conclusion

Our study demonstrated that Man had a protective effect against NAFLD through AMPK activation and NLRP3 inflammasome inhibition. Its treatment obviously improved body weight, liver weight/index, serum glucolipid metabolism, and insulin resistance and alleviated hepatic lipid accumulation and steatosis and liver function in HFD-induced NAFLD mice *in vivo*. In *in vitro* experiments, Man promoted hepatocyte glucose consumption and decreased FFA-induced intracellular TG and lipid accumulation. In addition, its protective mechanisms might be associated with the activity to regulate the AMPK and NLRP3 signaling pathways. Our findings will provide a scientific basis for the clinical application of Man to treat or precaution NAFLD in the future.

## Data Availability

The data used to support the findings of this study are included within the article.

## References

[1] Malaguarnera M., di Rosa M., Nicoletti F., Malaguarnera L. (2009). Molecular mechanisms involved in NAFLD progression. *Journal of Molecular Medicine*.

[2] Katzelnick L. C., Gresh L., Halloran M. E. (2017). Antibody-dependent enhancement of severe dengue disease in humans. *Science*.

[3] Younossi Z., Anstee Q. M., Marietti M. (2018). Global burden of NAFLD and NASH: trends, predictions, risk factors and prevention. *Nature Reviews Gastroenterology & Hepatology*.

[4] Buzzetti E., Pinzani M., Tsochatzis E. A. (2016). The multiple-hit pathogenesis of non-alcoholic fatty liver disease (NAFLD). *Metabolism*.

[5] Huang M., Kim H. G., Zhong X. (2020). Sestrin 3 protects against diet-induced nonalcoholic steatohepatitis in mice through suppression of transforming growth factor *β* signal transduction. *Hepatology*.

[6] Zhong X., Huang M., Kim H. G. (2020). SIRT6 protects against liver fibrosis by deacetylation and suppression of SMAD3 in hepatic stellate cells. *Cellular and Molecular Gastroenterology and Hepatology*.

[7] Chen S., Zhao X., Wan J. (2015). Dihydromyricetin improves glucose and lipid metabolism and exerts anti- inflammatory effects in nonalcoholic fatty liver disease: a randomized controlled trial. *Pharmacological Research*.

[8] Jazayeri-Tehrani S. A., Rezayat S. M., Mansouri S. (2019). Nano-curcumin improves glucose indices, lipids, inflammation, and Nesfatin in overweight and obese patients with non-alcoholic fatty liver disease (NAFLD): a double-blind randomized placebo-controlled clinical trial. *Nutrition & Metabolism*.

[9] Wang C., Jiang J. D., Wu W., Kong W. J. (2016). The compound of mangiferin-berberine salt has potent activities in modulating lipid and glucose metabolisms in HepG2 cells. *BioMed Research International*.

[10] Byrd C. M., Grosenbach D. W., Berhanu A. (2013). Novel benzoxazole inhibitor of dengue virus replication that targets the NS3 helicase. *Antimicrobial Agents and Chemotherapy*.

[11] Gaidhu M. P., Ceddia R. B. (2009). Remodeling glucose and lipid metabolism through AMPK activation: relevance for treating obesity and type 2 diabetes. *Clinical Lipidology*.

[12] Zhang B. B., Zhou G., Li C. (2009). AMPK: an emerging drug target for diabetes and the metabolic syndrome. *Cell Metabolism*.

[13] Day E. A., Ford R. J., Steinberg G. R. (2017). AMPK as a therapeutic target for treating metabolic diseases. *Trends in Endocrinology & Metabolism*.

[14] Ogawa Y., Imajo K., Honda Y. (2018). Palmitate-induced lipotoxicity is crucial for the pathogenesis of nonalcoholic fatty liver disease in cooperation with gut-derived endotoxin. *Scientific Reports*.

[15] Farrell G. C., Haczeyni F., Chitturi S. (2018). Pathogenesis of NASH: how metabolic complications of overnutrition favour lipotoxicity and pro-inflammatory fatty liver disease. *Obesity, Fatty Liver and Liver Cancer*.

[16] Wang Z., Zhang S., Xiao Y. (2020). NLRP3 inflammasome and inflammatory diseases. *Oxidative Medicine and Cellular Longevity*.

[17] di A., Xiong S., Ye Z. (2018). The TWIK2 potassium efflux channel in macrophages mediates NLRP3 inflammasome-induced inflammation. *Immunity*.

[18] Shi J., Zhao Y., Wang K. (2015). Cleavage of GSDMD by inflammatory caspases determines pyroptotic cell death. *Nature*.

[19] Yuan Y. Y., Wang S. L., Yuan L. W. (2018). Inflammatory caspase-related pyroptosis: mechanism, regulation and therapeutic potential for inflammatory bowel disease. *Gastroenterology Report*.

[20] Xu B., Jiang M., Chu Y. (2018). Gasdermin D plays a key role as a pyroptosis executor of non-alcoholic steatohepatitis in humans and mice. *Journal of Hepatology*.

[21] Wu J., Lin S., Wan B., Velani B., Zhu Y. (2019). Pyroptosis in liver disease: new insights into disease mechanisms. *Aging and Disease*.

[22] Sasdelli A. S., Brodosi L., Marchesini G. (2016). NAFLD-associated hepatocellular carcinoma: a threat to patients with metabolic disorders. *Current Hepatology Reports*.

[23] Saha S., Sadhukhan P., Sil P. C. (2016). Mangiferin: a xanthonoid with multipotent anti-inflammatory potential. *BioFactors*.

[24] Gold-Smith F., Fernandez A., Bishop K. (2016). Mangiferin and cancer: mechanisms of action. *Nutrients*.

[25] Yon C., Teramoto T., Mueller N. (2005). Modulation of the nucleoside triphosphatase/RNA helicase and 5′-RNA triphosphatase activities of dengue virus type 2 nonstructural protein 3 (NS3) by interaction with NS5, the RNA-dependent RNA polymerase. *The Journal of Biological Chemistry*.

[26] Na L., Zhang Q., Jiang S. (2015). Mangiferin supplementation improves serum lipid profiles in overweight patients with hyperlipidemia: a double-blind randomized controlled trial. *Scientific Reports*.

[27] Beier J. I., Banales J. M. (2018). Pyroptosis: an inflammatory link between NAFLD and NASH with potential therapeutic implications. *Journal of Hepatology*.

[28] Joshi-Barve S., Barve S. S., Amancherla K. (2007). Palmitic acid induces production of proinflammatory cytokine interleukin-8 from hepatocytes. *Hepatology*.

[29] Musso G., Cassader M., Gambino R. (2016). Non-alcoholic steatohepatitis: emerging molecular targets and therapeutic strategies. *Nature Reviews. Drug Discovery*.

[30] Pinky Gaidhu M., Ceddia R. B. (2009). Remodeling glucose and lipid metabolism through AMPK activation: relevance for treating obesity and type 2 diabetes. *Clinical Lipidology*.

[31] Chao H.-W., Chao S. W., Lin H., Ku H. C., Cheng C. F. (2019). Homeostasis of glucose and lipid in non-alcoholic fatty liver disease. *International Journal of Molecular Sciences*.

[32] Apontes P., Liu Z., Su K. (2014). Mangiferin stimulates carbohydrate oxidation and protects against high fat diet induced metabolic disorders. *Diabetes*.

[33] Shao Y., Yuan G., Zhang J., Guo X. (2015). Liraglutide reduces lipogenetic signals in visceral adipose of db/db mice with AMPK activation and Akt suppression. *Drug Design Development & Therapy*.

[34] Fritzen A. M., Lundsgaard A. M., Jordy A. B. (2015). New Nordic diet-induced weight loss is accompanied by changes in metabolism and AMPK signaling in adipose tissue. *Journal of Clinical Endocrinology & Metabolism*.

[35] De Nardo D., Latz E. (2011). NLRP3 inflammasomes link inflammation and metabolic disease. *Trends in Immunology*.

[36] Lebeaupin C., Proics E., de Bieville C. H. D. (2015). ER stress induces NLRP3 inflammasome activation and hepatocyte death. *Cell Death & Disease*.

[37] Wree A., McGeough M. D., Peña C. A. (2014). NLRP3 inflammasome activation is required for fibrosis development in NAFLD. *Journal of Molecular Medicine (Berlin, Germany)*.

[38] Angelico F., Del Ben M., Conti R. (2003). Insulin resistance, the metabolic syndrome and non alcoholic fatty liver disease (NAFLD). *Nutrition Metabolism & Cardiovascular Diseases*.

[39] Ikarashi N., Toda T., Okaniwa T., Ito K., Ochiai W., Sugiyama K. (2011). Anti-obesity and anti-diabetic effects of acacia polyphenol in obese diabetic KKAy mice fed high-fat diet. *Evidence-Based Complementary and Alternative Medicine*.

